# Supplementary material for: Loss of Peter Pan (PPAN) Affects Mitochondrial Homeostasis and Autophagic Flux
Source: Cells. 2019 Aug 14;8(8):894. doi: 10.3390/cells8080894 (PMC6721654; doi:10.3390/cells8080894)
Supplement: Supplementary file 1 [file cells-08-00894-s001.zip › proofed supplementary.docx]

Article

Loss of Peter Pan (PPAN) Affects Mitochondrial Homeostasis and Autophagic Flux

David P. Dannheisig ^1^, Eileen Beck ^1^, Enrico Calzia ^2^, Paul Walther ^3^, Christian Behrends ^4^ and Astrid S. Pfister ^1,^*

^1^ Institute of Biochemistry and Molecular Biology, Ulm University, D-89081 Ulm, Germany

^2^ Institute of Anesthesiological Pathophysiology and Process Development, Ulm University, D-89081 Ulm, Germany

^3^ Central Facility for Electron Microscopy, Ulm University, D-89081 Ulm, Germany

^4^ Munich Cluster for Systems Neurology, Medical Faculty, Ludwig-Maximilians-University München, D-81377 Munich, Germany

***** Correspondence: astrid.pfister@uni-ulm.de, Tel: +49 731 500 23390

**Supplemental Material**

**
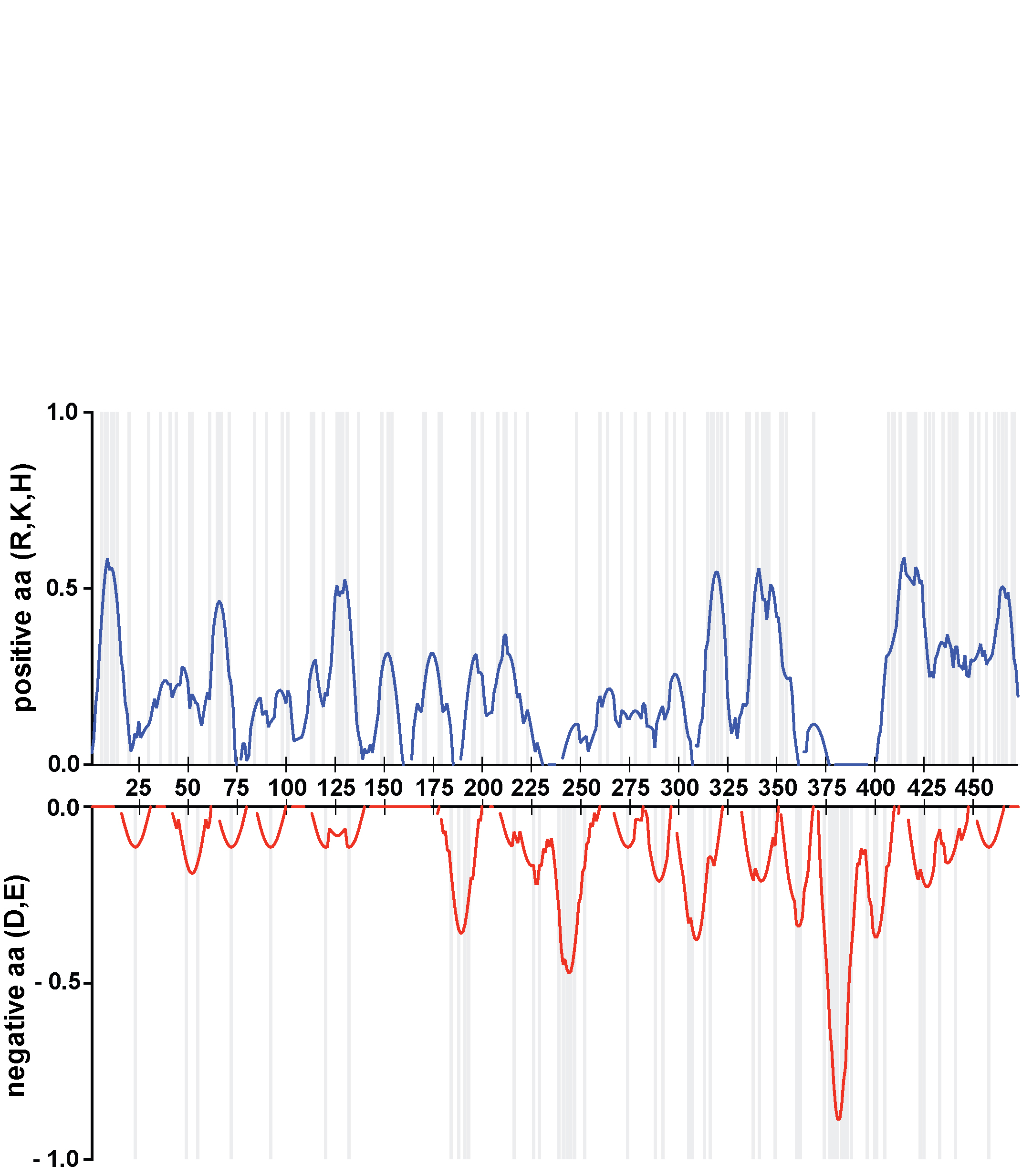
**

**Supplemental Figure 1: Charged amino acids within PPAN protein.** The PPAN protein sequence (Uniprot ID: Q9NQ55-1) analyzed with the EMBOSS Pepinfo (EMBL-EBI) using a hydropathy window size of 9. The figure was created with GraphPadPrism: Charged amino acids (aa) are highlighted by the grey bars: +1 denotes positively charged aa (R,K,H), and -1 negatively charged aa (D,E). The blue curve indicates positively charged residues (R,K,H) within a range of 10 adjacent aa on each side. The red curve indicates negatively charged residues (D,E) within a range of 10 adjacent aa on each side.


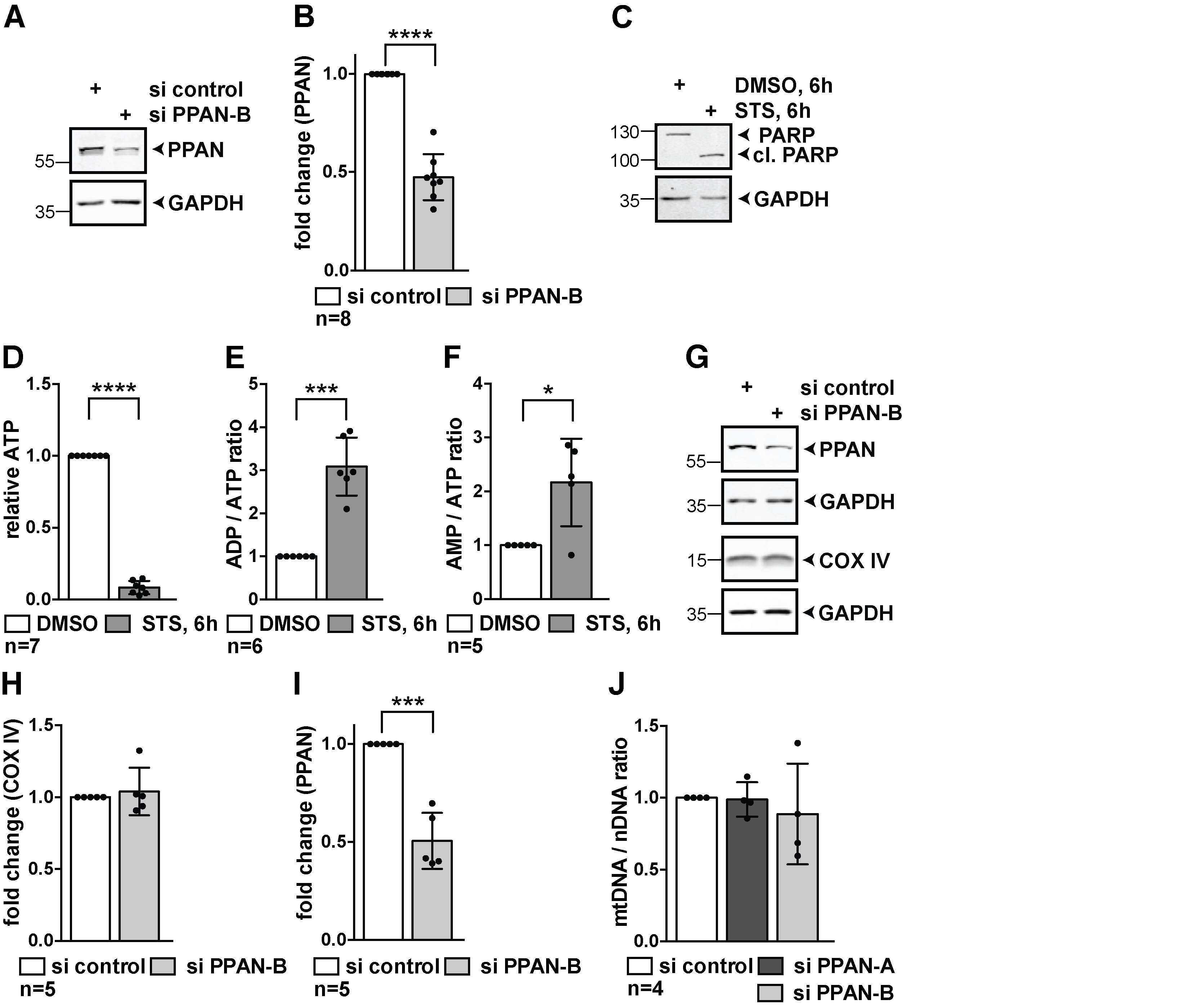


**Supplemental Figure 2: Control experiments related to Figure 2, STS control and effects of PPAN knockdown on COX IV protein and mtDNA / nDNA ratio.** (A,B) PPAN knockdown for 48 h in HeLa cells in 96-well format as indicated. (A) Whole cell lysates were subjected to Western blotting and membranes were probed with PPAN and GAPDH antibodies. (B) Quantification of PPAN normalized to GAPDH as shown in (A). The si control was set to 1. (C) Verification of STS-mediated apoptosis induction. Cells were treated in parallel to assays (D-F). Whole cell lysates were subjected to estern blotting and membranes were probed with PARP and GAPDH antibodies, cl. PARP denotes the caspase cleaved form of PARP, which is detected by the total PARP antibody. The Western blot is representative of nine experiments. (D-F) STS treatment reduces relative ATP levels and increases ADP / ATP and AMP / ATP ratio. HeLa cells were seeded in 96-well plates and treated with 330 nM staurosporine (STS) for 6 h to trigger apoptosis, and controls were treated with DMSO as indicated. ATP levels normalized to cell count, the ADP / ATP ratio and AMP / ATP ratio of STS and DMSO treated samples were detected by luminescence measurements. The DMSO control was set to 1. (G-I) PPAN knockdown for 48 h in HeLa cells (6-well format) does not affect COX IV protein levels. (G) Whole cell lysates were subjected to Western blotting and membranes were probed with COX IV, PPAN and GAPDH antibodies. Lysates of the same experiment were loaded twice (see GAPDH placed below respective blots). (H) Quantification of COX IV normalized to GAPDH as shown in (G), the differences are not significant. (I) Quantification of PPAN normalized to GAPDH as shown in (G). The si control was set to 1. (J) Measurement of mtDNA / nDNA ratio by qPCR analysis of HeLa cells transfected with control and PPAN siRNAs for 48 h as indicated, the differences are not significant The si control was set to 1. Error bars represent S.D.; p values were calculated by t‑test. Statistically significant differences are indicated by asterisks, *, p<0.05, ***, p<0.001, ****, p<0.0001; n= number of independent experiments.


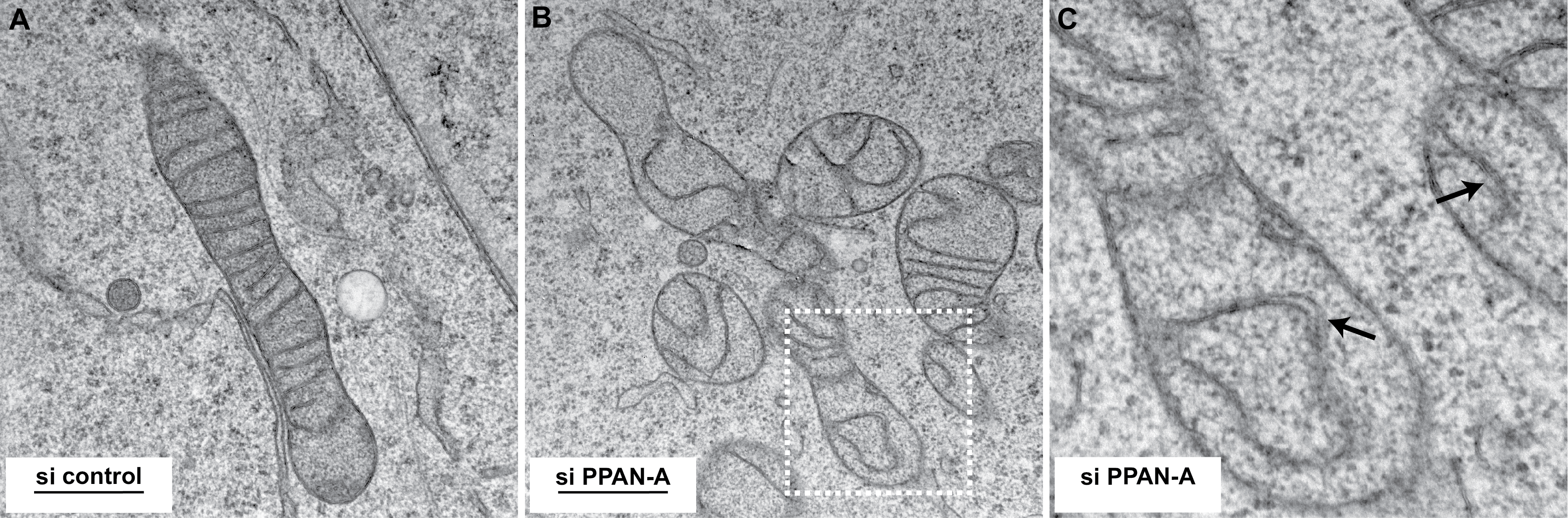


**Supplemental Figure 3: PPAN knockdown by si PPAN‑A abrogates mitochondrial architecture. (A)** HeLa cells were transiently transfected with control siRNA and cellular sections were analyzed for their mitochondrial phenotype by transmission electron microscopy (TEM). The assay is representative of two independent experiments. Scale bar, 1 µm. **(B)** HeLa cells were transfected with si PPAN‑A for 48 h. Representative cellular sections were imaged by TEM. Smaller mitochondria displaying impaired mitochondrial integrity and disrupted inner mitochondrial membranes following PPAN knockdown are shown. A representative image is shown, the TEM assay was performed two times. Scale bar, 1 µm. The boxed region is magnified in (C). **(C)** Magnification of the boxed area depicted in (B). The arrows point to perturbed cristae, indicated by a membranous character.


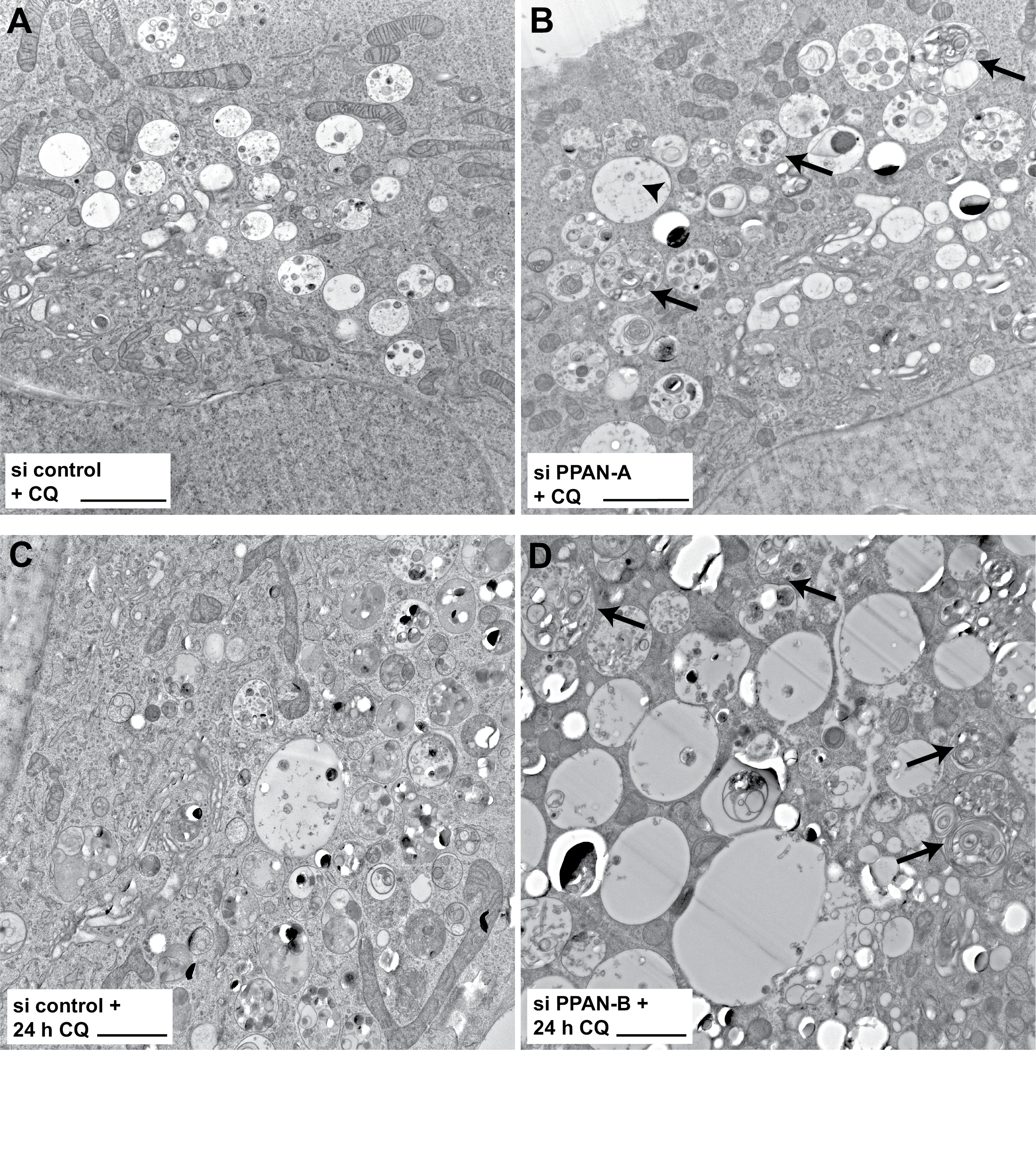


**Supplemental Figure 4: PPAN knockdown by si PPAN‑A enhances autophagosome accumulation in flux experiments as observed by transmission electron microscopy (TEM).** (A,B) HeLa cells were transfected with control siRNA (A) and si PPAN‑A for 48 h (B) and were incubated with CQ for 3.5 h, to block autophagic flux. Representative cell sections of two independent experiments are depicted. Scale bar, 2 µm. The arrows indicate accumulation of cargo-filled autophagosomes/-lysosomes, the arrowhead indicates expanded vesicles.


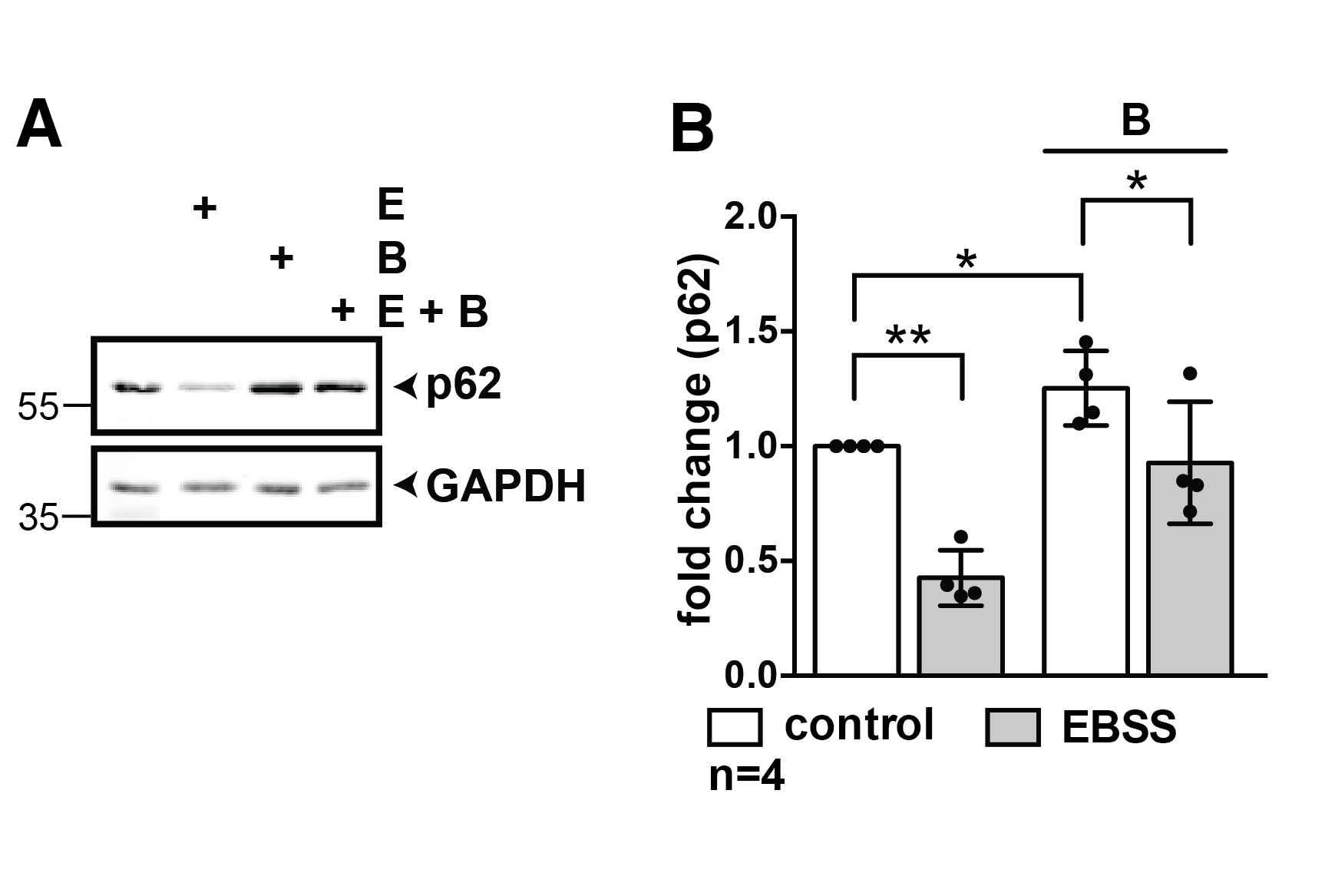


**Supplemental Figure 5: Control experiment for EBSS and BafA1 treatment in HeLa cells. (A,B)** Control experiment of EBSS-mediated starvation and BafA1-mediated flux block as determined by p62 turnover. Lysates of control transfected HeLa cells as shown in Figure 4 were subjected to Western blotting. Membranes were probed with p62 and GAPDH antibodies. (B) Statistical evaluation of p62 normalized to GAPDH from (A). The control was set to 1. Error bars represent S.D., p values were calculated by t‑test. Statistically significant differences are indicated by asterisks, *, p<0.05, **, p<0.01; n= number of independent experiments.


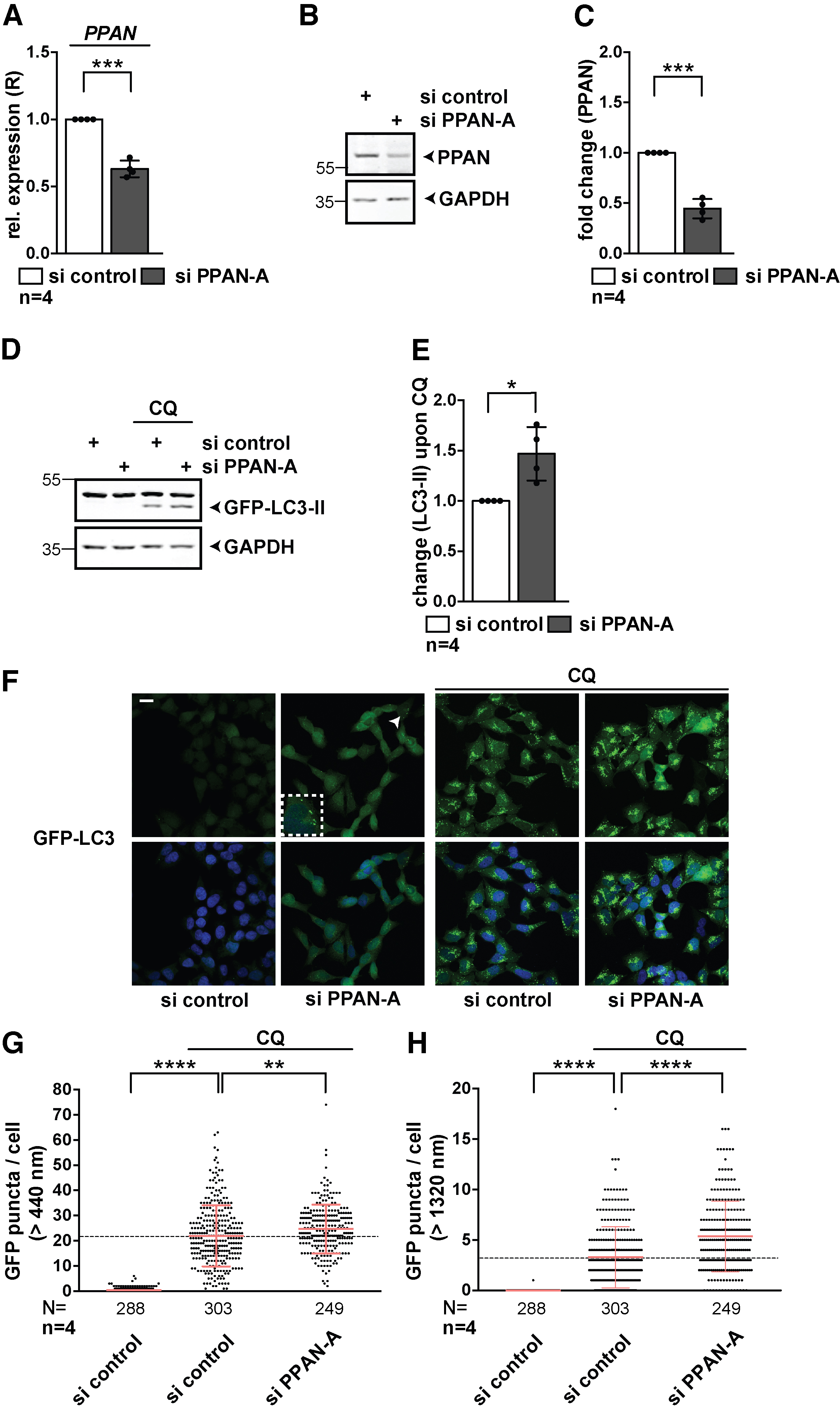


**Supplemental Figure 6: PPAN knockdown by si PPAN‑A enhances basal autophagy in HEK293A GFP‑LC3 cells. (A)** qPCR analysis of HEK293A GFP‑LC3 cells transfected with siRNAs as indicated. Transfection of si PPAN‑A results in significant down-regulation of *PPAN* normalized to *GAPDH* (=relative expression, R). **(B,C)** PPAN knockdown in HEK293A GFP‑LC3 cells as indicated. Western blots of hypotonic lysates were probed with PPAN and GAPDH antibodies, the statistical evaluation is shown in (C). **(D,E)** GFP‑LC3‑II levels increase after PPAN knockdown by si PPAN‑A in HEK293A GFP‑LC3 cells in presence of CQ for 3.5 h. Western blots of whole cell lysates were probed with total LC3 and GAPDH antibodies, the statistical evaluation of the flux is shown in (E). The si control was set to 1 as indicated. Error bars represent S.D., p values were calculated by t‑test (in A,C,E). Statistically significant differences are indicated by asterisks, *, p<0.05, ***, p<0.001; n= number of independent experiments. **(F)** PPAN knockdown by si PPAN‑A increases accumulation of GFP‑LC3 puncta in HEK293A GFP‑LC3 cells. Representative confocal image stacks of randomly chosen optical fields are depicted, the upper panels show the GFP fluorescence, and the lower panels the merge with DAPI. For flux experiments, cells were treated with CQ for 3.5 h. Scale bar, 2 µm. The arrowhead points to representative GFP‑LC3 puncta in PPAN knockdown samples, which are magnified in the boxed inset. **(G,H)** Random image stacks of (F) were counted for GFP‑LC3 positive puncta per cell, indicating autophagosome accumulation. The mean of all counted cells transfected and treated as indicated is shown. In (G) all puncta larger than 440 nm, in (H) puncta larger than 1320 nm were included for analysis. Of note, si control values are the same as in Figure 5, since si PPAN‑B and si PPAN‑A samples were simultaneously analyzed. Error bars represent S.D., p values were calculated by unpaired t‑test (in G,H). Statistically significant differences are indicated by asterisks, **, p<0.01, ****, p<0.0001; N= number of independently counted cells, n= number of independent experiments used for single cell counting.

| 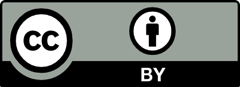 | © 2019 by the authors. Submitted for possible open access publication under the terms and conditions of the Creative Commons Attribution (CC BY) license (http://creativecommons.org/licenses/by/4.0/). |
| --- | --- |
